# 2,4-Diiodo-3-nitro­anisole

**DOI:** 10.1107/S160053681200952X

**Published:** 2012-04-21

**Authors:** Xianfei Li, Lei Cao, Chuansheng Ruan, Baoming Ji, Le Zhou

**Affiliations:** a College of Science, Northwest Agriculture and Forest University, Yangling 712100, People’s Republic of China; b College of Life Science, Northwest Agriculture and Forest University, Yangling 712100, People’s Republic of China; c Zhengzhou University, People’s Republic of China; d College of Chemistry and Chemical Engineering, Luoyang Normal University, Luoyang 471022, People’s Republic of China

## Abstract

In the title compound (systematic name: 1,3-diiodo-4-meth­oxy-2-nitro­benzene), C_7_H_5_I_2_NO_3_, the dihedral angle between the benzene ring and the nitro group is 88.0 (3)°, and the methyl group lies almost in the same plane as the ring [deviation = 0.034 (6) Å]. In the crystal, aromatic π–π stacking occurs between inversion-related rings [centroid–centroid separation = 3.865 (3) Å and slippage = 0.642 Å]. A possible weak C—I⋯π inter­action occurs [I⋯π = 3.701 (2) Å and C—I⋯π = 130.18 (13)°], but there are no significant inter­molecular I⋯I contacts.

## Related literature

For the crystal structures of isomers of the title compound, see: Garden *et al.* (2002 ▶, 2004 ▶).
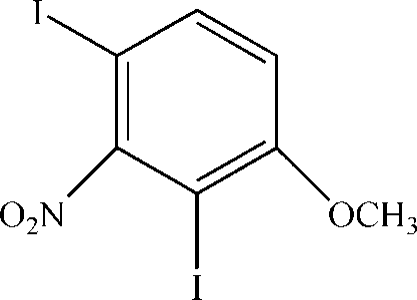


## Experimental

### 

#### Crystal data

C_7_H_5_I_2_NO_3_
*M* *_r_* = 404.92
Monoclinic, 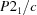

*a* = 9.264 (2) Å
*b* = 8.756 (2) Å
*c* = 13.549 (3) Å
β = 108.835 (2)°
*V* = 1040.2 (4) Å^3^
*Z* = 4
Mo *K*α radiation
μ = 6.02 mm^−1^
*T* = 296 K
0.36 × 0.33 × 0.14 mm

#### Data collection

Bruker SMART CCD diffractometer
Absorption correction: multi-scan (*SADABS*; Bruker, 1998 ▶) *T*_min_ = 0.220, *T*_max_ = 0.486
7459 measured reflections
1937 independent reflections
1712 reflections with *I* > 2σ(*I*)
*R*_int_ = 0.018

#### Refinement

*R*[*F* ^2^ > 2σ(*F* ^2^)] = 0.027
*wR*(*F* ^2^) = 0.065
*S* = 1.00
2689 reflections
172 parameters
H-atom parameters constrained
Δρ_max_ = 1.06 e Å^−3^
Δρ_min_ = −1.25 e Å^−3^

### 

Data collection: *SMART* (Bruker, 1998 ▶); cell refinement: *SAINT* (Bruker, 1998 ▶); data reduction: *SAINT*; program(s) used to solve structure: *SHELXS97* (Sheldrick, 2008 ▶); program(s) used to refine structure: *SHELXL97* (Sheldrick, 2008 ▶); molecular graphics: *SHELXTL* (Sheldrick, 2008 ▶); software used to prepare material for publication: *SHELXTL*.

## Supplementary Material

Crystal structure: contains datablock(s) global, I. DOI: 10.1107/S160053681200952X/hb6662sup1.cif

Structure factors: contains datablock(s) I. DOI: 10.1107/S160053681200952X/hb6662Isup2.hkl

Supplementary material file. DOI: 10.1107/S160053681200952X/hb6662Isup3.cml

Additional supplementary materials:  crystallographic information; 3D view; checkCIF report

## Figures and Tables

**Fig. 1.**
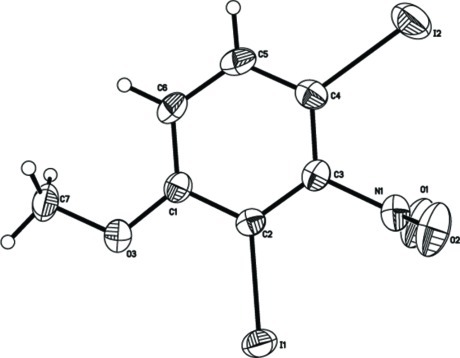
The moleuclar sturcture of (I) with displacement ellipsoids drawn at the 30% probability level.

**Fig. 2.**
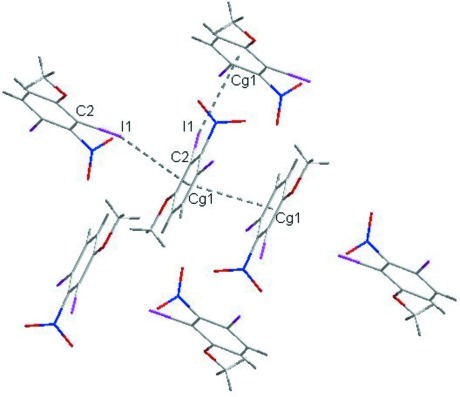
Part of the crystal structure of the title compound, showing formation of a sheet built from π···π stacking interactions, and C—I···π interactions.

## supplementary crystallographic information

### Comment

We report here the molecular and supramolecular structures of the title compound, (I), which is isomeric with 2,6-diiodo-4-nitroanisole (Garden *etal.*, 2002) and 2,4-diiodo-6-nitroanisole (Garden *et al.*, 2003). The changed position of iodo, nitro and methoxy may lead to different interactions such as iodo-nitro interactions, and aromatic π···π stacking interactions.

The asymmetric unit of the title compound comprise a whole molecule of 2,4-diiodo-3-nitroanisole (Fig. 1). Atoms I1, I2, C7 and O3 are almost coplanar with the benzene ring. On the contrary, the plane defined by the nitro group is almost perpendicular to the plane of the aromatic ring and form a dihedral angle of 88.0 (4)°. In contrast with 2,6-diiodo-4-nitroanisole (Garden *et al.*, 2002) and 2,4-diiodo-6-nitroanisole (Garden *et al.*, 2003), there is no iodo-nitro interaction in the compound, each molecule link three others by π···π stacking interaction and C—I···π interaction, leading to the formation of a sheet (Fig. 2). The aryl ring planes (centroid *Cg*1) of two molecules are parallel, show a π···π stacking interaction *Cg*1···*Cg*1^viii^ [symmetry codes: (viii) 1 - *x*, 2 - *y*, -*z*), and the centroid distance is 3.865 (3) Å. C—I···π interaction also occurs in the compound, I1 aim to the phenyl ring [I1···*Cg*1^ix^ 3.701 (2) Å, C2—I1···*Cg*1^ix^ 130.1 (1)°; symmetry code: (ix) 1 - *x*, 1/2 + *y*, 1/2 - *z*].

### Experimental

The title compound was obtained from 2-iodo-3-nitrophenol, a solution of 2-iodo-3-nitrophenol (2 mmol) in acetone (20 ml) was added K_2_CO_3_ (5 mmol). The mixture was stirred at room temperature for 30 min, then CH_3_I (5 mmol) was added at once. The resulting solution was then stirred at 343 K for 3 h. The addition of ice (20 g) prompted the precipitation of the title compound, which was collected by filtration and crystallized from ethyl acetate as yellow blocks (yield 90%, m.p. 406–408 K).

### Refinement

All H atoms were located from difference maps and were treated as riding atoms with C—H distances of 0.93 Å (aromatic) and 0.96 Å (methyl).

### Crystal data

**Table:**

C_7_H_5_I_2_NO_3_	*F*(000) = 736
*M**_r_* = 404.92	*D*_x_ = 2.586 Mg m^−^^3^
Monoclinic, *P*2_1_/*c*	Mo *K*α radiation, λ = 0.71073 Å
*a* = 9.264 (2) Å	Cell parameters from 3452 reflections
*b* = 8.756 (2) Å	θ = 2.8–27.0°
*c* = 13.549 (3) Å	µ = 6.02 mm^−^^1^
β = 108.835 (2)°	*T* = 296 K
*V* = 1040.2 (4) Å^3^	Block, yellow
*Z* = 4	0.36 × 0.33 × 0.14 mm

### Data collection

**Table:**

Bruker SMART CCD diffractometer	1937 independent reflections
Radiation source: fine-focus sealed tube	1712 reflections with *I* > 2σ(*I*)
Graphite monochromator	*R*_int_ = 0.018
phi and ω scans	θ_max_ = 25.5°, θ_min_ = 2.3°
Absorption correction: multi-scan (*SADABS*; Bruker, 1998)	*h* = −11→11
*T*_min_ = 0.220, *T*_max_ = 0.486	*k* = −10→10
7459 measured reflections	*l* = −16→16

### Refinement

**Table:**

Refinement on *F*^2^	Primary atom site location: structure-invariant direct methods
Least-squares matrix: full	Secondary atom site location: difference Fourier map
*R*[*F*^2^ > 2σ(*F*^2^)] = 0.027	Hydrogen site location: inferred from neighbouring sites
*wR*(*F*^2^) = 0.065	H-atom parameters constrained
*S* = 1.00	*w* = 1/[σ^2^(*F*_o_^2^) + (0.0264*P*)^2^ + 3.0364*P*] where *P* = (*F*_o_^2^ + 2*F*_c_^2^)/3
2689 reflections	(Δ/σ)_max_ = 0.001
172 parameters	Δρ_max_ = 1.06 e Å^−^^3^
0 restraints	Δρ_min_ = −1.25 e Å^−^^3^

### Special details

**Table:**

Geometry. All esds (except the esd in the dihedral angle between two l.s. planes) are estimated using the full covariance matrix. The cell esds are taken into account individually in the estimation of esds in distances, angles and torsion angles; correlations between esds in cell parameters are only used when they are defined by crystal symmetry. An approximate (isotropic) treatment of cell esds is used for estimating esds involving l.s. planes.
Refinement. Refinement of F^2^ against ALL reflections. The weighted R-factor wR and goodness of fit S are based on F^2^, conventional R-factors R are based on F, with F set to zero for negative F^2^. The threshold expression of F^2^ > 2sigma(F^2^) is used only for calculating R-factors(gt) etc. and is not relevant to the choice of reflections for refinement. R-factors based on F^2^ are statistically about twice as large as those based on F, and R- factors based on ALL data will be even larger.

### Fractional atomic coordinates and isotropic or equivalent isotropic displacement parameters (Å^2^)

**Table:**

	*x*	*y*	*z*	*U*_iso_*/*U*_eq_	
C1	0.3646 (5)	0.8010 (5)	0.0510 (3)	0.0431 (10)	
C2	0.4466 (5)	0.8963 (5)	0.1324 (3)	0.0391 (9)	
C3	0.6036 (5)	0.9031 (5)	0.1575 (3)	0.0405 (9)	
C4	0.6821 (5)	0.8208 (6)	0.1041 (3)	0.0465 (11)	
C5	0.5990 (6)	0.7285 (6)	0.0233 (3)	0.0519 (12)	
H5	0.6490	0.6724	−0.0141	0.062*	
C6	0.4412 (6)	0.7182 (6)	−0.0030 (3)	0.0488 (11)	
H6	0.3870	0.6549	−0.0574	0.059*	
C7	0.1252 (6)	0.7011 (7)	−0.0503 (4)	0.0659 (15)	
H7A	0.1457	0.7238	−0.1138	0.099*	
H7B	0.0185	0.7152	−0.0607	0.099*	
H7C	0.1529	0.5971	−0.0306	0.099*	
N1	0.6918 (5)	1.0000 (5)	0.2475 (3)	0.0507 (10)	
O1	0.7319 (6)	0.9427 (5)	0.3316 (3)	0.0860 (14)	
O2	0.7193 (5)	1.1291 (5)	0.2286 (3)	0.0831 (13)	
O3	0.2116 (4)	0.8002 (4)	0.0299 (2)	0.0571 (9)	
I1	0.33063 (4)	1.02858 (4)	0.21050 (3)	0.05654 (12)	
I2	0.91883 (4)	0.82973 (6)	0.14145 (3)	0.08415 (18)	

### Atomic displacement parameters (Å^2^)

**Table:**

	*U*^11^	*U*^22^	*U*^33^	*U*^12^	*U*^13^	*U*^23^
C1	0.048 (2)	0.046 (2)	0.034 (2)	−0.002 (2)	0.0103 (18)	0.0012 (19)
C2	0.047 (2)	0.037 (2)	0.034 (2)	0.0034 (19)	0.0143 (18)	0.0028 (18)
C3	0.048 (2)	0.039 (2)	0.032 (2)	−0.0018 (19)	0.0096 (18)	0.0047 (18)
C4	0.044 (2)	0.056 (3)	0.041 (2)	0.007 (2)	0.0163 (19)	0.009 (2)
C5	0.067 (3)	0.054 (3)	0.040 (2)	0.009 (2)	0.024 (2)	−0.002 (2)
C6	0.062 (3)	0.049 (3)	0.033 (2)	0.000 (2)	0.011 (2)	−0.0057 (19)
C7	0.054 (3)	0.093 (4)	0.046 (3)	−0.021 (3)	0.009 (2)	−0.017 (3)
N1	0.050 (2)	0.056 (3)	0.043 (2)	−0.0101 (19)	0.0105 (18)	0.0048 (18)
O1	0.119 (4)	0.081 (3)	0.039 (2)	−0.024 (3)	−0.001 (2)	0.005 (2)
O2	0.105 (3)	0.064 (3)	0.065 (2)	−0.034 (2)	0.007 (2)	0.000 (2)
O3	0.0453 (18)	0.070 (2)	0.0505 (19)	−0.0050 (16)	0.0083 (15)	−0.0121 (17)
I1	0.0635 (2)	0.0536 (2)	0.0582 (2)	0.00317 (16)	0.02756 (16)	−0.01141 (15)
I2	0.0470 (2)	0.1373 (4)	0.0684 (3)	0.0114 (2)	0.01898 (18)	−0.0001 (2)

### Geometric parameters (Å, º)

**Table:**

C1—O3	1.353 (5)	C5—C6	1.391 (7)
C1—C6	1.378 (6)	C5—H5	0.9300
C1—C2	1.396 (6)	C6—H6	0.9300
C2—C3	1.384 (6)	C7—O3	1.419 (6)
C2—I1	2.086 (4)	C7—H7A	0.9600
C3—C4	1.382 (6)	C7—H7B	0.9600
C3—N1	1.493 (6)	C7—H7C	0.9600
C4—C5	1.379 (7)	N1—O1	1.189 (5)
C4—I2	2.087 (5)	N1—O2	1.205 (5)
			
O3—C1—C6	124.7 (4)	C6—C5—H5	119.6
O3—C1—C2	115.9 (4)	C1—C6—C5	120.6 (4)
C6—C1—C2	119.4 (4)	C1—C6—H6	119.7
C3—C2—C1	118.7 (4)	C5—C6—H6	119.7
C3—C2—I1	121.6 (3)	O3—C7—H7A	109.5
C1—C2—I1	119.6 (3)	O3—C7—H7B	109.5
C4—C3—C2	122.5 (4)	H7A—C7—H7B	109.5
C4—C3—N1	118.9 (4)	O3—C7—H7C	109.5
C2—C3—N1	118.6 (4)	H7A—C7—H7C	109.5
C5—C4—C3	117.9 (4)	H7B—C7—H7C	109.5
C5—C4—I2	119.2 (3)	O1—N1—O2	125.2 (4)
C3—C4—I2	122.9 (3)	O1—N1—C3	117.6 (4)
C4—C5—C6	120.8 (4)	O2—N1—C3	117.2 (4)
C4—C5—H5	119.6	C1—O3—C7	117.3 (4)
			
O3—C1—C2—C3	−179.6 (4)	C3—C4—C5—C6	−0.4 (7)
C6—C1—C2—C3	−1.0 (6)	I2—C4—C5—C6	179.2 (4)
O3—C1—C2—I1	−1.1 (5)	O3—C1—C6—C5	178.8 (4)
C6—C1—C2—I1	177.5 (3)	C2—C1—C6—C5	0.3 (7)
C1—C2—C3—C4	1.0 (6)	C4—C5—C6—C1	0.4 (7)
I1—C2—C3—C4	−177.5 (3)	C4—C3—N1—O1	−90.3 (6)
C1—C2—C3—N1	−177.4 (4)	C2—C3—N1—O1	88.1 (6)
I1—C2—C3—N1	4.2 (5)	C4—C3—N1—O2	88.1 (6)
C2—C3—C4—C5	−0.3 (7)	C2—C3—N1—O2	−93.5 (5)
N1—C3—C4—C5	178.0 (4)	C6—C1—O3—C7	3.3 (7)
C2—C3—C4—I2	−179.9 (3)	C2—C1—O3—C7	−178.2 (4)
N1—C3—C4—I2	−1.5 (6)		
